# Medial bicompartmental arthroplasty patients display more normal gait and improved satisfaction, compared to matched total knee arthroplasty patients

**DOI:** 10.1007/s00167-021-06773-8

**Published:** 2021-10-23

**Authors:** Amy J. Garner, Oliver W. Dandridge, Richard J. van Arkel, Justin P. Cobb

**Affiliations:** 1 MSk Lab, Sir Michael Uren Biomedical Engineering Research Hub, Imperial College London, White City Campus, 80-92 Wood Lane, London, W12 0BZ UK; 2 Biomechanics Group, Mechanical Engineering Department, Imperial College London, London, SW7 1AZ UK; 3 Royal College of Surgeons of England and Dunhill Medical Trust Clinical Research Fellowship, Royal College of Surgeons of England, 35-43 Lincoln’s Inn Fields, London, WC2A 3PE UK; 4 Health Education Kent, Surrey and Sussex, Stewart House, 32 Russell Square, London, WC1B 5DN UK

**Keywords:** Gait, Bicompartmental knee arthroplasty, Compartmental arthroplasty, Total knee arthroplasty, Walking speed, Satisfaction, Medial with patellofemoral arthroplasty

## Abstract

**Purpose:**

Medial bicompartmental arthroplasty, the combination of ipsilateral medial unicompartmental and patellofemoral arthroplasty, is an alternative to total knee arthroplasty for patients with medial tibiofemoral and severe patellofemoral arthritis, when the lateral tibiofemoral compartment and anterior cruciate ligament are intact. This study reports the gait and subjective outcomes following medial bicompartmental arthroplasty.

**Methods:**

Fifty-five subjects were measured on the instrumented treadmill at top walking speeds, using standard metrics of gait. Modular, single-stage, medial bicompartmental arthroplasty subjects (*n* = 16) were compared to age, body mass index, height- and sex-matched healthy (*n* = 19) and total knee arthroplasty (*n* = 20) subjects. Total knee arthroplasty subjects with pre-operative evidence of tricompartmental osteoarthritis or anterior cruciate ligament dysfunction were excluded. The vertical component of ground reaction force and temporospatial measurements were compared using Kruskal–Wallis, then Mann–Whitney test with Bonferroni correction (*α* = 0.05). Oxford Knee and EuroQoL EQ-5D scores were compared.

**Results:**

Objectively, the medial bicompartmental arthroplasty top walking speed of 6.7 ± 0.8 km/h was 0.5 km/h (7%) slower than that of healthy controls (*p* = 0.2), but 1.3 km/h (24%) faster than that of total knee arthroplasty subjects (5.4 ± 0.6 km/h, *p* < 0.001). Medial bicompartmental arthroplasty recorded more normal maximum weight acceptance (*p* < 0.001) and mid-stance forces (*p* = 0.03) than total knee arthroplasty subjects, with 11 cm (15%) longer steps (*p* < 0.001) and 21 cm (14%) longer strides (*p* = 0.006). Subjectively, medial bicompartmental arthroplasty subjects reported Oxford Knee Scores of median 41 (interquartile range 38.8–45.5) compared to total knee arthroplasty Oxford Knee Scores of 38 (interquartile range 30.5–41, *p* < 0.02). Medial bicompartmental arthroplasty subjects reported EQ-5D median 0.88 (interquartile range 0.84–0.94) compared to total knee arthroplasty median 0.81 (interquartile range 0.73–0.89, *p* < 0.02.)

**Conclusion:**

This study finds that, in the treatment of medial tibiofemoral osteoarthritis with severe patellofemoral arthritis, medial bicompartmental arthroplasty results in nearer-normal gait and improved patient-reported outcomes compared to total knee arthroplasty.

**Level of evidence:**

III.

**Supplementary Information:**

The online version contains supplementary material available at 10.1007/s00167-021-06773-8.

## Introductions

Total knee arthroplasty (TKA) remains the gold-standard treatment for osteoarthritis (OA) of the knee, with widely reported success. Relative to TKA, unicompartmental knee arthroplasty (UKA) is associated with more normal gait [21, 35], higher satisfaction [26] and fewer peri- and post-operative serious complications [25], but significantly higher revision rates [1, 23]. There is limited evidence for multi-compartment PKA.

**Fig. 1:**
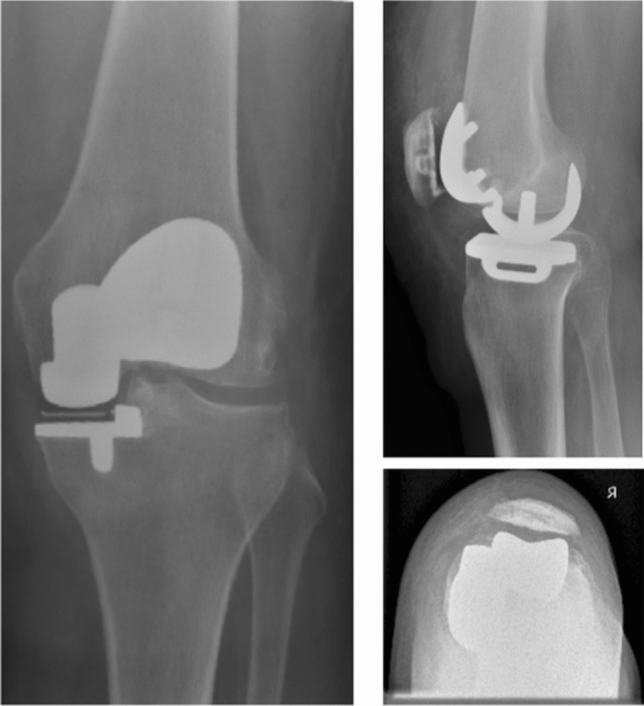
Radiographs depicting a medial bicompartmental arthroplasty: left: anterior–posterior view, top right: lateral view, bottom right: skyline view

Of those undergoing primary knee arthroplasty, 23% have medial tibiofemoral and patellofemoral (PFJ) OA, with a spared lateral compartment [31]. Medial bicompartmental arthroplasty (BCA-M), the combination of ipsilateral medial UKA and patellofemoral arthroplasty (PFA), is a bone, meniscus and anterior cruciate ligament (ACL)-preserving alternative to TKA (Fig. 1) [12, 14]. This study seeks to understand the gait characteristics and patient-reported outcomes of BCA-M compared to patients treated with a posterior- cruciate retaining TKA. The null hypothesis is that there are no differences between BCA-M and TKA in these parameters.

## Materials and methods

A previous instrumented treadmill study compared the top walking speed of UKA-M (*n* = 12) to healthy controls (*n* = 121) and TKA (*n* = 12) [21]. UKA TWS was 7.9 km/h, similar to healthy subjects (TWS 7.9 km/h) but 37% faster than TKA (5.76 km/h). We assumed that the TKA subjects in our study would not differ significantly from previous studies, but predicted that BCA-M may walk up to 15% faster (TWS 6.63 km/h, 15% faster). A power calculation indicated that a minimum of 14 subjects per implant group would be necessary to detect such differences with 80% power and 95% confidence.

**Fig. 2:**
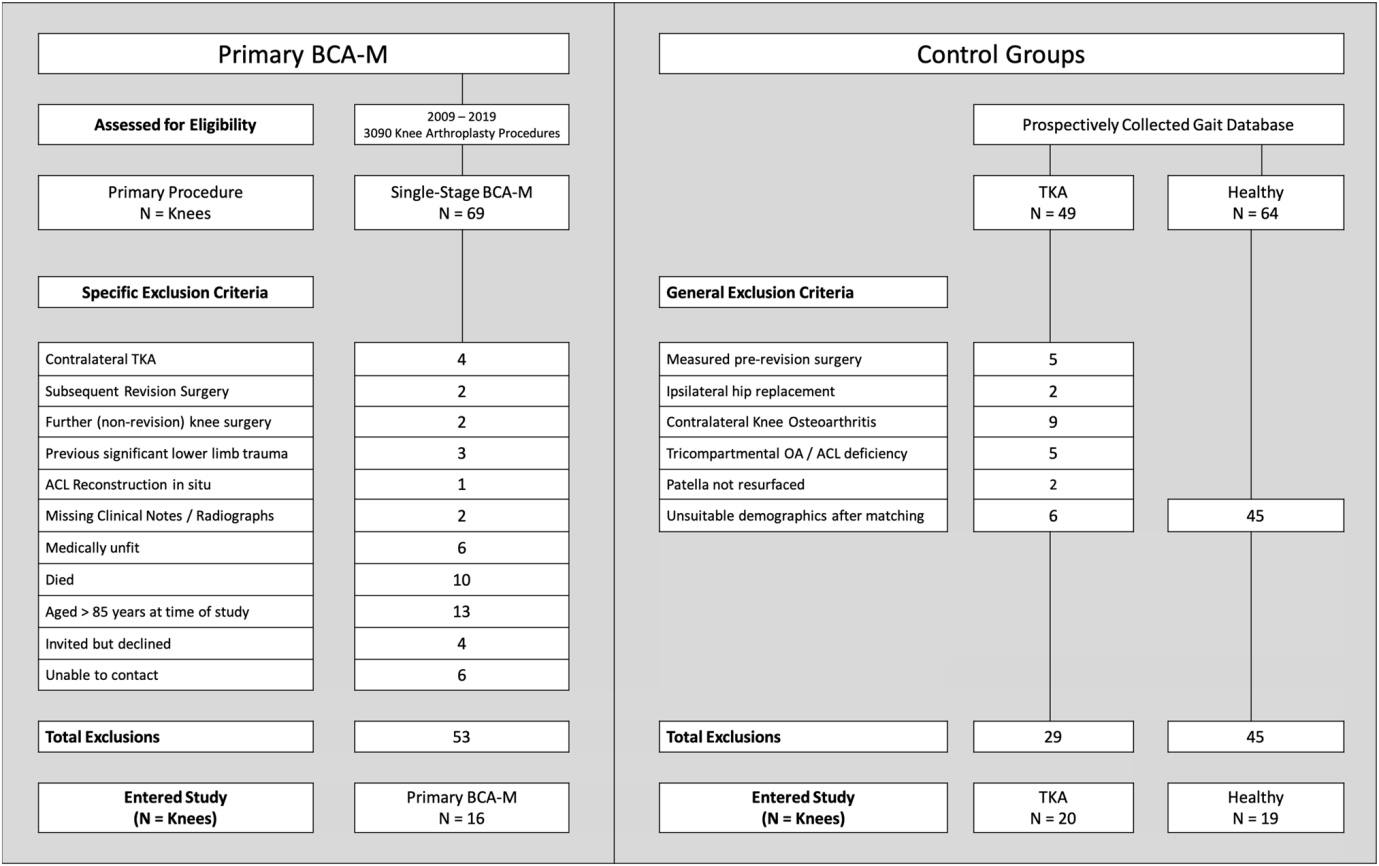
Pathway to entry into the study for medial bicompartmental arthroplasty (BCA-M), posterior-cruciate retaining total knee arthroplasty (TKA) and healthy subjects

Potential subjects were retrospectively identified from the operative database of the senior author, between 2009 and 2019. Of 3090 knee arthroplasty procedures, 69 patients had undergone primary modular BCA-M. Subjects were excluded if they had a contralateral TKA in situ (*n* = 4, Fig. 2, Supplementary Table A); if they had undergone significant ipsilateral limb surgery pre- or post-BCA-M (*n* = 8), which included two subjects who had been revised following BCA-M; were over 85 years (*n* = 13); medically unfit (*n* = 6) or deceased (*n* = 10); or if they declined (*n* = 4) or were uncontactable (*n* = 6). After 53 exclusions (Fig. 2, Supplementary Table A), 16 BCA-M patients entered the study.

### Matching

With institutional review board approval from NRES Committee South Central and the Academic Health Science Centre, Imperial College London and Imperial College Healthcare NHS Trust, UK, our group prospectively evaluated healthy subjects and the patients of three senior arthroplasty surgeons on an instrumented treadmill (Kistler Gaitway, Kistler Instrument Corporation, Amherst, NY), over the past 9 years. Data were collected by a research assistant blinded to arthroplasty status. For the current study, the database was searched to identify suitable control subjects for comparison to the BCA-M group. At the time of data analysis, 19 healthy subjects matched to the age, body mass index (BMI) and sex of the BCA-M group (Table 1, Fig. 2). Forty-nine posterior-cruciate retaining TKA subjects were identified in the database. Potential TKA subjects were excluded if they had been measured prior to scheduled revision surgery (*n* = 5); had an ipsilateral hip replacement in situ (*n* = 2); had significant OA of the contralateral knee (*n* = 9); tricompartmental OA graded Kellgren and Lawrence (KL) ≥ 2 or evidence of ACL deficiency (*n* = 5), defined by > 7 mm anterior tibial translation on pre-operative radiographs [9]. The remaining TKA subjects underwent age, sex and body mass index matching (IBM^®^ SPSS^®^ Version 27, Table 1) resulting in 22 potential posterior-cruciate retaining TKA subjects. Since all BCA-M subjects had undergone patellar resurfacing, a further two TKA subjects were excluded, since they had not undergone patellar resurfacing. Consequently, 20 posterior-cruciate retaining TKA subjects entered the study, of whom 11 had single-compartment disease (medial *n* = 8, lateral *n* = 3). The remaining had two-compartment disease (medial and lateral *n* = 8, lateral with patellofemoral *n* = 1). TKAs were mean 40.6 ± 43 months post-surgery, 19.6 months longer than primary BCA-M (21 ± 18) though this did not reach statistical significance (*p* = 0.1). The median months post-surgery were BCA-M 12.5 months (range 6–65 months) compared to TKA, 19.5 months (range 6–147 months, *p* = 0.5).

**Table 1:** Demographics, gait characteristics at top walking speeds and patient-reported outcomes of primary medial bicompartmental arthroplasty (BCA-M) subjects compared to healthy controls and total knee arthroplasty (TKA) subjects

Subject	Healthy	Primary BCA-M	TKA
Number of knees (*n* =)	19	16	20
Sex: M:F (% male)	7: 12 (37%)	6: 10 (38%)	7: 13 (35%)
Age (years)	63 ± 9.7	68 ± 8.1	65 ± 9.9
Body mass index (kg/m^2^)	26.6 ± 4.7	26.9 ± 5.1	27.2 ± 3.9
Height (cm)	171 ± 10	175 ± 8	171 ± 12
Mean months post-surgery (SD)		21 ± 18	40.6 ± 43
Median months post-surgery (range)		12.5 (6–65)	19.5 (6–147)
Top walking speed (km/h)	7.2 ± 0.7^b^	6.7 ± 0.8^c^	5.4 ± 0.6^b,c^
Hof speed (H)	0.73 ± 0.1^b^	0.67 ± 0.1^c^	0.54 ± 0.1^b,c^
Weight acceptance rate (BW/s)	10.2 ± 3.3	9.0 ± 3.0	7.5 ± 3.3
Maximum weight acceptance force (BW)	1.6 ± 0.2^b^	1.4 ± 0.2^c^	1.2 ± 0.1^b,c^
Mid-stance force (BW)	0.5 ± 0.1^a,b^	0.6 ± 0.2^a,c^	0.7 ± 0.1^b,c^
Push-off force (BW)	1.0 ± 0.1	1.0 ± 0.1	1.0 ± 0.1
Push-off rate (BW/s)	4.0 ± 0.9	3.8 ± 1.0	3.8 ± 0.8
Step length (cm)	82 ± 8^b^	82 ± 8^c^	71 ± 7^b,c^
Stride length (cm)	165 ± 17^b^	164 ± 15^c^	144 ± 17^b,c^
Gait width (cm)	12 ± 3	12 ± 3	14 ± 3
Cadence (step/min)	60 ± 4.9^b^	58 ± 4.4^c^	52 ± 4.4^b,c^
Impulse (BW/s)	382 ± 23	379 ± 30	385 ± 25
Double support time (s)	0.29 ± 0.05	0.27 ± 0.05^c^	0.34 ± 0.08^c^
Contact time (s)	1.29 ± 0.1^b^	1.31 ± 0.2^c^	1.50 ± 0.2^b,c^
OKS mean (range)		41.4 ± 5^c^	36.0 ± 6.5^c^
OKS median (interquartile range)		41 (38.8–45.5)^c^	38 (30.5–41)^c^
EQ-5D mean		0.89 ± 0.07^c^	0.77 ± 0.16^c^
EQ-5D median (interquartile range)		0.88 (0.84–0.94)^c^	0.81 (0.73–0.89)^c^

All values are means with standard deviations unless otherwise stated. Sex and median months post-surgery are each compared with a Chi-square test, and all other demographics compared with ANOVA. Gait variables were subjected to Kruskall–Wallis then Mann–Whitney test with Bonferroni correction where significant differences were found. All tests, significance *p* value < 0.05

*BW* normalized to body weight

^a^Healthy vs. BCA-M < 0.05

^b^Healthy vs. TKA *p* < 0.05

^c^BCA-M Vs. TKA *p* < 0.05

Where no superscript is noted, *p* > 0.05

### Treadmill testing

All subjects walked at 4 km/h for 2 min to acclimatize to the treadmill, before increasing speed in 0.5 km/h increments to their ‘top walking speed’ (TWS) defined as their fastest comfortable speed, or the highest walking speed before breaking into a run. Subjects walked, on average, for 12 min continuously, without the assistance of the hand safety rail. All subjects completed the test comfortably. Two tandem force plates, beneath the moving belt, recorded the vertical component of the ground reaction forces, temporospatial measurements and centre of pressure for both limbs, sampling at 100 Hz frequency over 10 s. To correct for differences in leg length and body mass, data were normalized post-collection using Hof scaling [20] and normalization of body weight (BW = ground reaction force/(body mass/gravity)) respectively.

### Patient-reported outcome measures

Arthroplasty subjects were asked to complete the Oxford Knee Score (OKS) and EuroQoL EQ-5D 5L Score at the time of their treadmill assessment.

### Statistical analysis

TKA and healthy subjects were matched to the BCA-M cohort in IBM^®^ SPSS^®^ Version 27 for age (*p* = 0.3), sex (*p* = 1), body mass index (*p* = 0.9) and height (*p* = 0.5) from the prospectively collected database (Table 1). Gait output data were averaged using a custom MathWorks^®^ MatLab^®^ script and analysed in IBM^®^ SPSS^®^ Version 27. The Shapiro–Wilk test demonstrated that a number of variables were not normally distributed; therefore, all variables were compared using Kruskal–Wallis, then Mann–Whitney test with Bonferroni correction where differences were detected. Significance was set at *α* = 0.05.

## Results

### Top walking speeds

**Fig. 3:**
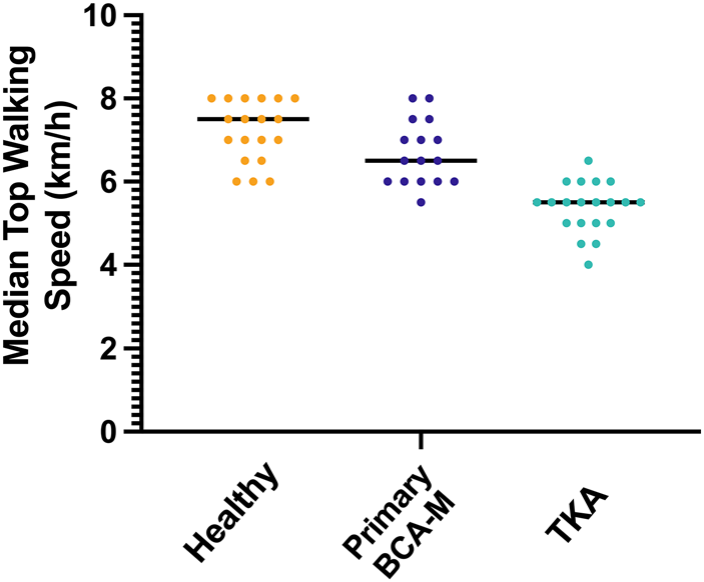
Median top walking speed (km/h) for primary medial bicompartmental arthroplasty (BCA-M) compared to healthy subjects and posterior-cruciate retaining total knee arthroplasty (TKA)

Objectively, the medial bicompartmental arthroplasty top walking speed of 6.7 ± 0.8 km/h was 0.5 km/h (7%) slower than that of healthy controls (*p* = 0.16, Table 1, Fig. 3), but 1.3 km/h (24%) faster than that of total knee arthroplasty subjects (5.4 ± 0.6 km/h, *p* < 0.001). The TKA group walked 25% slower than healthy subjects (*p* < 0.001). The differences remained apparent after Hof scaling for leg length (Table 1).

### Vertical ground reaction forces

**Fig. 4:**
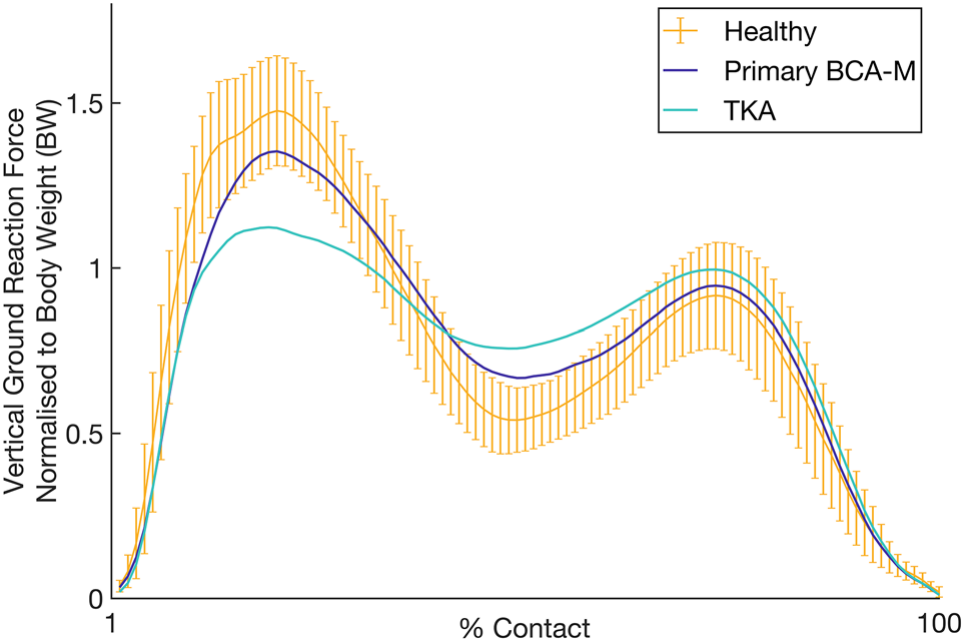
Vertical ground reaction force normalized for body weight during stance phase of gait for subjects with a medial bicompartmental arthroplasty (BCA-M) compared to primary posterior-cruciate retaining total knee arthroplasty (TKA). Normal range for healthy subjects shown with 95% confidence intervals

At top walking speeds, compared to healthy subjects, both arthroplasty groups recorded reduced weight acceptance, though the differences did not reach significance (*p* > 0.05, Table 1, Fig. 4). BCA-M and healthy subjects recorded similar maximum weight acceptance force (*p* = 0.051), whilst in TKA subjects it was reduced (*p* < 0.001). During mid-stance, both groups recorded higher forces than the healthy cohort (*p* < 0.03); however, the BCA-M subjects were nearer normal, with a significant advantage over the TKA group (*p* < 0.03). All groups were similar in terms of push-off force and rate (*p* = 1). Compared to healthy and BCA-M subjects, TKA subjects recorded reduced cadence (*p* < 0.003, Table 1), increased double support time (Vs BCA-M *p* = 0.03) and increased contact time (*p* < 0.003). BCA-M subjects displayed nearer-normal temporospatial characteristics, with differences failing to reach significance when compared to healthy controls.

### Step and stride lengths

**Fig. 5:**
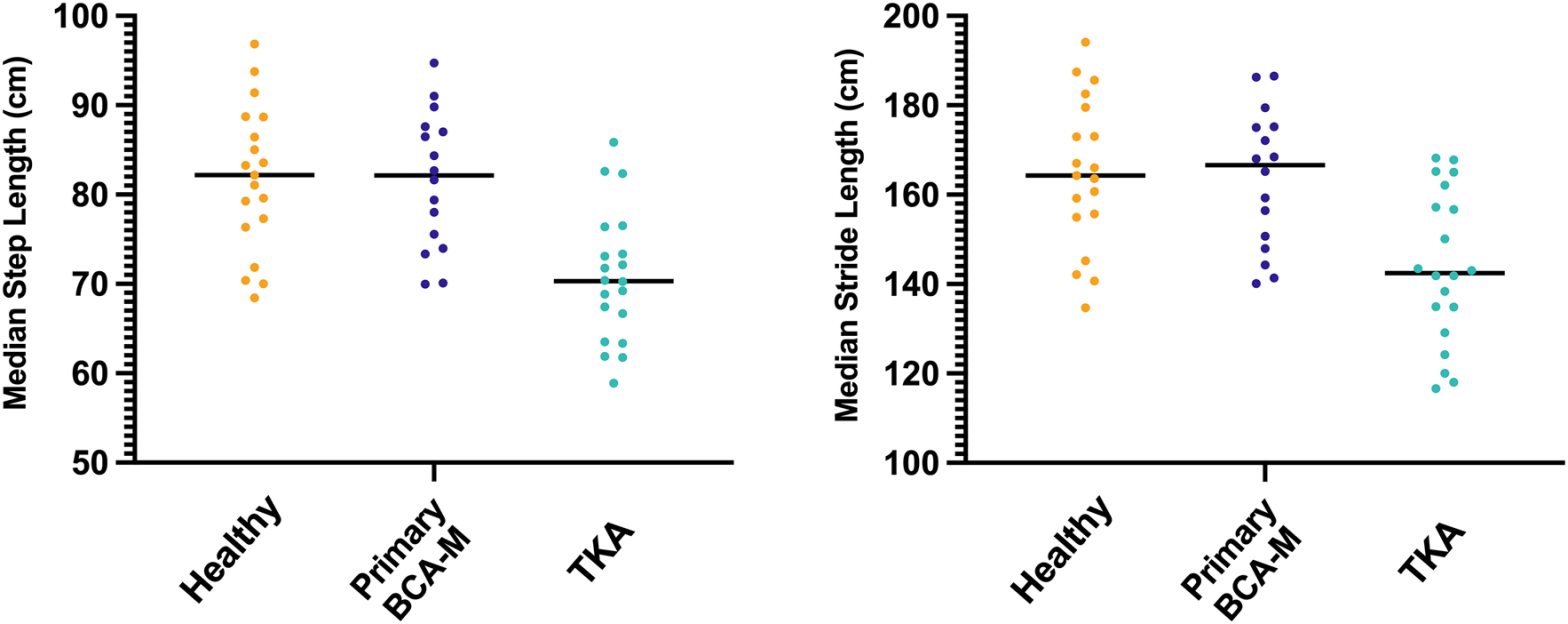
Median step length (left) and stride length (right) for primary medial bicompartmental arthroplasty (BCA-M) compared to matched healthy controls and posterior-cruciate retaining total knee arthroplasty (TKA)

BCA-M subjects’ median step lengths were similar to those of healthy subjects (both 82 cm), whilst those of TKA subjects were median 12 cm (15%) shorter (Fig. 5). This reflected the differences in stride length, whereby median BCA-M stride lengths were 167 cm (IQR 149-175 cm), 3 cm (2%) longer than those of healthy subjects, while TKA stride lengths were median 142 cm, 22 cm (13%) shorter than those of healthy subjects (Fig. 5).

### Satisfaction and quality of life

**Fig. 6:**
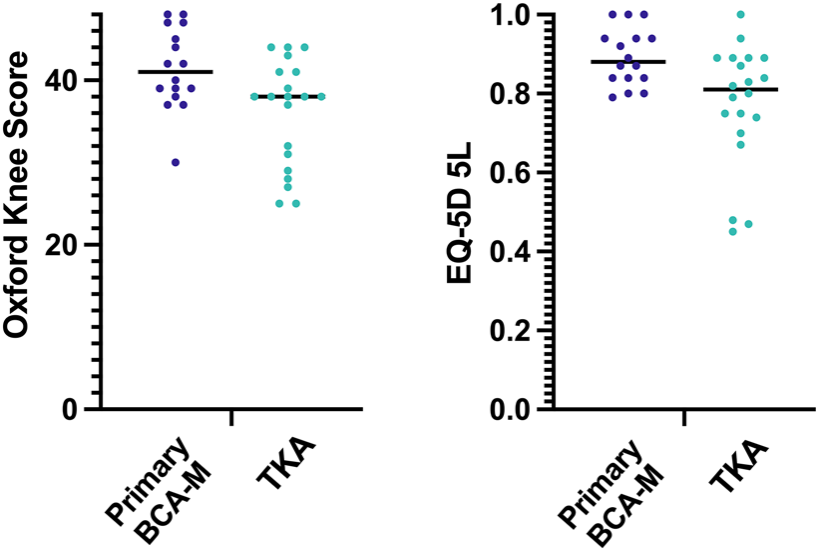
Median Oxford Knee score and EuroQol-5D (EQ-5D) for medial bicompartmental arthroplasty (BCA-M) compared to total knee arthroplasty (TKA)

OKS and EQ-5D scores were completed by all arthroplasty subjects. For the OKS, median scores were significantly higher for BCA-M than TKA (41 vs 38, *p* = 0.02, Fig. 6, Table 1). Though not validated by individual question, it is noteworthy that BCA-M scored equal to or higher than TKA in all questions of the OKS, with significant differences seen in the use of transport, chair rising, kneeling, instability symptoms and stair descent (all *p* < 0.03, Table 2). Similarly, median EQ-5D values were higher for BCA-M compared to TKA (0.88 vs 0.81. *p* < 0.02, Fig. 6, Table 1). BCA-M subjects recorded scores closer to 1 in every domain, reaching significance in mobility, usual activities and pain (*p* < 0.02, Table 3).

**Table 2:** Oxford Knee Scores between BCA-M and TKA groups by overall score, where 48 is the best possible outcome and by individual question

	BCA-M	TKA	Difference	p value
Overall OKS	41.4 ± 5	36.0 ± 6.5	5.4	**0.016**
Q1. How would you describe the pain you usually have on your knee?	2.8 ± 1.1	2.8 ± 1.1	0	1
Q2. Have you had any trouble with washing and drying yourself (all over) because of your knee?	3.8 ± 0.5	3.8 ± 0.4	0.1	0.4
Q3. Have you had any trouble getting in or out of a car or using public transport because of your knee?	3.3 ± 0.8	2.6 ± 1.0	0.7	**0.02**
Q4. For how long have you been able to walk before pain from your knee becomes severe?	3.8 ± 0.5	3.4 ± 0.9	0.5	0.07
Q5. How painful has it been for you to stand up from a chair because of your knee?	3.4 ± 0.8	2.8 ± 0.7	0.6	**0.01**
Q6. Have you been limping when walking because of your knee?	3.6 ± 0.6	3.1 ± 1.0	0.5	0.2
Q7. Could you kneel down and get up again afterwards?	2.8 ± 1.2	1.6 ± 1.0	1.3	**0.002**
Q8. Have you been troubled by pain from your knee in bed at night?	3.3 ± 0.8	3.2 ± 1.0	0.1	0.9
Q9. How much has pain from your knee interfered with your usual work?	3.6 ± 0.5	3.1 ± 0.8	0.3	0.8
Q10. Have you felt that your knee may suddenly 'give way' or let you down?	3.6 ± 0.5	3.1 ± 0.8	0.6	**0.03**
Q11. Could you do household shopping on your own?	3.9 ± 0.3	3.4 ± 1.3	0.5	0.3
Q12. Could you walk down one flight of stairs?	3.7 ± 0.8	3.1 ± 0.9	0.6	**0.005**

Each question has a maximum score of 4 for the best outcome. Values shown are means with standard deviation. Significant differences Mann–Whitney test *p* < 0.05 highlighted in bold

**Table 3:** EuroQol 5D Scores between BCA-M and TKA groups by overall score and by individual domain

	BCA-M	TKA	Difference	*p* Value
Overall EQ5D	0.89 ± 0.07	0.77 ± 0.16	0.12	**0.012**
Mobility	1.5 ± 0.7	2.5 ± 1.0	0.95	**0.004**
Self-care	1.1 ± 0.3	1.4 ± 0.7	0.34	0.07
Usual activities	1.6 ± 0.7	2.6 ± 1.1	0.99	**0.005**
Pain	1.9 ± 0.6	2.4 ± 0.7	0.53	**0.02**
Anxiety	1.2 ± 0.4	1.3 ± 0.7	0.11	0.8

The best possible outcome for the overall score is 1. Each domain is graded 1–5 where 1 is the best overall outcome. Values shown are means with standard deviation. Significant differences Mann–Whitney test *p* < 0.05 highlighted in bold

## Discussion

This study finds that BCA-M has advantages over TKA in terms of gait and patient-reported outcomes, leading to the null hypothesis being rejected.

Severe lateral facet PFJ OA is considered by many to be a relative contraindication to medial UKA [4, 5], leading to TKA in the majority of these patients [1, 23]. In those with isolated medial with patellofemoral wear, TKA necessitates resection of healthy lateral bone, menisci, and a functional ACL. BCA-M may be considered appropriate for those with medial tibiofemoral and severe patellofemoral arthrosis (KL ≥ 2), with correctable varus, anterior–posterior sagittal stability and medial collateral ligament sufficiency (Table 4).

**Table 4:** Indications and contraindications for primary medial bicompartmental arthroplasty

Indications	Contraindications
Medial with patellofemoral arthrosis Kellgren and Lawrence Score ≥ 2	Lateral tibiofemoral arthrosis Kellgren and Lawrence Score ≥ 2
Spared lateral compartment (Kellgren and Lawrence Score < 2)	Medial collateral ligament disruption/dysfunction/significant laxity
Functional anterior cruciate ligament*	Anterior cruciate ligament dysfunction*
Correctable varus	Inflammatory arthropathy

*ACL dysfunction in the elderly is a relative contraindication, provided that the knee is otherwise stable

BCA-M has documented clinical success in the short and medium term [11, 19, 30] and is thought to restore isokinetic quadriceps function [33], whilst preserving near-native extensor efficiency [13]. There is some evidence that BCA-M results in higher function during strenuous activity [28], but no studies to our knowledge have compared BCA-M to healthy controls and TKA at top walking speeds. Arthroplasty performance can be objectively assessed using gait analysis on the instrumented treadmill. Previous studies have reported an advantage of UKA-M over TKA at top walking speeds [18, 21, 35] which the current study suggests is preserved following primary modular BCA-M, most notably in top walking speeds and stride length [21, 35]. Previous BCA-M gait studies have focused on historic monolithic BCA-M designs, some of which were blighted by high revision rates. These studies included fewer BCA-M subjects walking at slower walking speeds to those investigated here [24, 34].

The marked difference in top walking speed between BCA-M and TKA is particularly important. For every 0.1 m per second increase in top walking speed, life expectancy improves significantly and may be considered a proxy measure for a subject’s global post-operative health [32]. BCA-M is more anterior–posterior stable than TKA [15] where ACL resection can result in paradoxical anterior–posterior tibial translation, limiting mid-swing flexion and impacting stride length and walking speed [22]. Unlike TKA, BCA-M preserves extensor efficiency at the low flexion angles associated with gait [13], which may in part explain why differences are seen during maximum weight acceptance and mid-stance when the quadriceps are active.

Survivorship of primary BCA-M is largely unknown. The National Joint Registry has reported that primary multi-compartmental arthroplasty has a similar revision rate to primary patellofemoral arthroplasty, though the numbers are small and include all compartmental combinations, not just BCA-M [2]. Of the 69 primary BCA-M subjects considered for this study, two knees (3%) had been revised (Fig. 2, Supplemental Table A), one to a tricompartmental arthroplasty through the addition of a lateral UKA (Revision Partial Knee Classification PR2b [17]), 7 years after primary BCA-M, and one to a posterior-cruciate retaining TKA (Revision Partial Knee Classification PR3), performed at another hospital for unexplained pain after 4 years. Of the subjects included in the study, no subjects have since been revised.

Progressive arthritis remains a common mode of failure following single-compartment PKA [2, 6, 7]. The ‘compartmental approach’ (Revision Partial Knee Classification PR2b [17]) advocates the addition of a second PKA to the newly degenerate compartment, while retaining the primary PKA and ACL [14, 18]. It has been shown to preserve healthy gait characteristics, despite second surgery [16], reflecting the results of the present study.

The study was powered for gait analysis, with OKS and EQ-5D 5L scores collected as secondary outcome measures, though the study was underpowered for PROMs. Overall satisfaction and quality of life was good after both BCA-M and TKA, but a statistical advantage was reported following BCA-M. The difference in mean OKS exceeded the reported minimal important clinical difference of five points [3], though the difference in median OKS scores did not (Table 1). This suggests that there may be an advantage in PROMs with BCA-M, compared to this relatively satisfied cohort of TKAs. The recorded OKS and EQ-5D scores are similar to widely reported literature values for UKA and TKA [8, 26, 36], with no apparent adverse effect as a consequence of the additional PFA in BCA-M subjects when compared to medial UKA, supporting the findings of others [29]. Higher satisfaction is also reported in those revised through a compartmental approach [16]. The significant differences observed on the OKS in relation to rising from a chair, kneeling down, stair descent and instability symptoms support the theory that BCA-M preserves isokinetic quadriceps strength and anterior–posterior stability, known to be compromised following TKA [13, 15, 27].

This study would have benefited from pre-operative data to determine the extent of improvement each subject experienced as a consequence of surgery. However, attempts were made to mitigate its absence by only selecting TKA patients who underwent surgery for single- or two-compartment disease, did not have pre-operative evidence of ACL dysfunction, and would have been eligible for PKA or CPKA under the senior author’s current clinical practice. The inclusion of patients with single-compartmental disease in the TKA group was necessary to power the study, but may have acted to the detriment of the BCA-M group, who all had bicompartmental disease pre-operatively.

### Clinical relevance

The retention of the lateral compartment and the cruciate ligament complex may play an important role in allowing patients with a BCA-M to retain near normal stride length and speed following surgery. The data may help clinicians and their patients when deciding on alternatives to TKA where once the benefits may have been considered unsubstantiated [10].

## Conclusions

This study finds that, in the treatment of medial tibiofemoral osteoarthritis with severe patellofemoral arthritis, medial bicompartmental arthroplasty results in nearer-normal gait and improved patient-reported outcomes compared to total knee arthroplasty.

## Supplementary Information

Below is the link to the electronic supplementary material.

Supplementary file1 (DOCX 15 KB)

## References

[1] Ben-Shlomo Y, Blom A, Boulton C, Brittain R, Clark E, Craig R et al (2019) National joint registry annual reports. The national joint registry 16th annual report 2019. National Joint Registry, London 32744812

[2] (2021) 18th annual report. National Joint Registry 35072993

[3] Beard DJ, Harris K, Dawson J, Doll H, Murray DW, Carr AJ (2015). Meaningful changes for the Oxford hip and knee scores after joint replacement surgery. J Clin Epidemiol.

[4] Beard DJ, Pandit H, Gill HS, Hollinghurst D, Dodd CA, Murray DW (2007). The influence of the presence and severity of pre-existing patellofemoral degenerative changes on the outcome of the Oxford medial unicompartmental knee replacement. J Bone Jt Surg Br.

[5] Beard DJ, Pandit H, Ostlere S, Jenkins C, Dodd CA, Murray DW (2007). Pre-operative clinical and radiological assessment of the patellofemoral joint in unicompartmental knee replacement and its influence on outcome. J Bone Jt Surg Br.

[6] Berger RA, Meneghini RM, Jacobs JJ, Sheinkop MB, Valle CJD, Rosenberg AG (2005). Results of unicompartmental knee arthroplasty at a minimum of ten years of follow-up. J Bone Jt Surg Am.

[7] Berger RA, Meneghini RM, Sheinkop MB, Della Valle CJ, Jacobs JJ, Rosenberg AG (2004). The progression of patellofemoral arthrosis after medial unicompartmental replacement: results at 11 to 15 years. Clin Orthop Relat Res.

[8] Burn E, Sanchez-Santos MT, Pandit HG, Hamilton TW, Liddle AD, Murray DW (2018). Ten-year patient-reported outcomes following total and minimally invasive unicompartmental knee arthroplasty: a propensity score-matched cohort analysis. Knee Surg Sports Traumatol Arthrosc.

[9] Chiu SS (2006). The anterior tibial translocation sign. Radiology.

[10] Cobb JP (2014). Patient safety after partial and total knee replacement. Lancet.

[11] Confalonieri N, Manzotti A, Cerveri P, De Momi E (2009). Bi-unicompartmental versus total knee arthroplasty: a matched paired study with early clinical results. Arch Orthop Trauma Surg.

[12] Garner A, Cobb J, Rivière C, Vendittoli P-A (2020). Combined partial knee arthroplasty. Personalized hip and knee joint replacement.

[13] Garner A, Dandridge O, Amis AA, Cobb JP, van Arkel RJ (2021). The extensor efficiency of unicompartmental, bicompartmental, and total knee arthroplasty. Bone Jt Res.

[14] Garner A, van Arkel RJ, Cobb J (2019). Classification of combined partial knee arthroplasty. Bone Jt J.

[15] Garner AJ, Dandridge OW, Amis AA, Cobb JP, van Arkel RJ (2021). Partial and combined partial knee arthroplasty: greater anterior-posterior stability than posterior cruciate-retaining total knee arthroplasty. J Arthroplasty.

[16] Garner AJ, Dandridge OW, van Arkel RJ, Cobb JP (2021). The compartmental approach to revision of partial knee arthroplasty results in nearer-normal gait and improved patient reported outcomes compared to total knee arthroplasty. Knee Surg Sports Traumatol Arthrosc.

[17] Garner AJ, Edwards TC, Liddle AD, Jones GG, Cobb JP (2021). The revision partial knee classification system: understanding the causative pathology and magnitude of further surgery following partial knee arthroplasty. Bone Jt Open.

[18] Haddad FS, Masri BA (2019). Compartmental arthroplasty: time for a clear nomenclature. Bone Jt J.

[19] Heyse TJ, Khefacha A, Cartier P (2010). UKA in combination with PFR at average 12-year follow-up. Arch Orthop Trauma Surg.

[20] Hof AL (1996). Scaling gait data to body size. Gait Posture.

[21] Jones GG, Kotti M, Wiik AV, Collins R, Brevadt MJ, Strachan RK (2016). Gait comparison of unicompartmental and total knee arthroplasties with healthy controls. Bone Jt J.

[22] Kirtley C, Whittle MW, Jefferson RJ (1985). Influence of walking speed on gait parameters. J Biomed Eng.

[23] Klasan A, Parker DA, Lewis PL, Young SW (2021). Low percentage of surgeons meet the minimum recommended unicompartmental knee arthroplasty usage thresholds: analysis of 3037 Surgeons from Three National Joint Registries. Knee Surg Sports Traumatol Arthrosc.

[24] Leffler J, Scheys L, Plante-Bordeneuve T, Callewaert B, Labey L, Bellemans J (2012). Joint kinematics following bi-compartmental knee replacement during daily life motor tasks. Gait Posture.

[25] Liddle AD, Judge A, Pandit H, Murray DW (2014). Adverse outcomes after total and unicompartmental knee replacement in 101,330 matched patients: a study of data from the National Joint Registry for England and Wales. Lancet.

[26] Liddle AD, Pandit H, Judge A, Murray DW (2015). Patient-reported outcomes after total and unicompartmental knee arthroplasty: a study of 14,076 matched patients from the National Joint Registry for England and Wales. Bone Jt J.

[27] Lundberg HJ, Rojas IL, Foucher KC, Wimmer MA (2016). Comparison of antagonist muscle activity during walking between total knee replacement and control subjects using unnormalized electromyography. J Arthroplasty.

[28] Palumbo BT, Henderson ER, Edwards PK, Burris RB, Gutierrez S, Raterman SJ (2011). Initial experience of the Journey-Deuce bicompartmental knee prosthesis: a review of 36 cases. J Arthroplasty.

[29] Parratte S, Ollivier M, Opsomer G, Lunebourg A, Argenson JN, Thienpont E (2015). Is knee function better with contemporary modular bicompartmental arthroplasty compared to total knee arthroplasty? Short-term outcomes of a prospective matched study including 68 cases. Orthop Traumatol Surg Res.

[30] Parratte S, Pauly V, Aubaniac JM, Argenson JN (2010). Survival of bicompartmental knee arthroplasty at 5 to 23 years. Clin Orthop Relat Res.

[31] Stoddart JC, Dandridge O, Garner A, Cobb J, Arkel R (2020). The compartmental distribution of knee osteoarthritis—a systematic review and meta-analysis. Osteoarthr Cartil.

[32] Studenski S, Perera S, Patel K, Rosano C, Faulkner K, Inzitari M (2011). Gait speed and survival in older adults. JAMA.

[33] Thienpont E, Price A (2013). Bicompartmental knee arthroplasty of the patellofemoral and medial compartments. Knee Surg Sports Traumatol Arthrosc.

[34] Wang H, Foster J, Franksen N, Estes J, Rolston L (2018). Gait analysis of patients with an off-the-shelf total knee replacement versus customized bi-compartmental knee replacement. Int Orthop.

[35] Wiik AV, Manning V, Strachan RK, Amis AA, Cobb JP (2013). Unicompartmental knee arthroplasty enables near normal gait at higher speeds, unlike total knee arthroplasty. J Arthroplasty.

[36] Williams DP, Blakey CM, Hadfield SG, Murray DW, Price AJ, Field RE (2013). Long-term trends in the Oxford knee score following total knee replacement. Bone Jt J.

