# Supplementary material for: Medial bicompartmental arthroplasty patients display more normal gait and improved satisfaction, compared to matched total knee arthroplasty patients
Source: Knee Surg Sports Traumatol Arthrosc. 2021 Oct 23;31(3):830–8. doi: 10.1007/s00167-021-06773-8 (PMC9958162; doi:10.1007/s00167-021-06773-8)
Supplement: Supplementary file 1 — Supplementary file1 (DOCX 15 KB) [file 167_2021_6773_MOESM1_ESM.docx]

Supplementary Table A. Reasons for exclusion of primary BCA-M subjects from the study.

| **Reason for Exclusion** | **N=** | **Details** |
| --- | --- | --- |
| Missing Clinical Notes / Radiographs | 2 |  |
| Contralateral Total Knee Arthroplasty in situ | 4 |  |
| ACL Reconstruction in Situ | 1 |  |
| Subsequent Revision Surgery | 1 | Conversion to Tricompartmental Arthroplasty after 5yrs (RPKC class PR2b) |
|  | 1 | Revision to TKA after 7 months (another centre, RPKC class PR3) |
| Further ipsilateral (non-revision) knee surgery | 1 | Medial Patella-femoral Ligament Reconstruction (RPKC class PR1) |
|  | 1 | Open Reduction Internal Fixation Proximal Tibia for peri-prosthetic fracture |
| Previous significant trauma | 1 | Femoral Open Reduction Internal Fixation |
|  | 1 | Ankle Fracture Open Reduction Internal Fixation with residual pain |
|  | 1 | Contralateral calcaneal fracture with residual discomfort on fast walking |
|  | 1 | Anterior Cruciate Ligament Reconstruction in situ |
| Medically Unfit | 4 | Significant cardiac disease |
|  | 2 | Significant pulmonary disease |
|  | 1 | Recent surgery for colorectal cancer |
|  | 1 | Parkinson’s Disease |
| Died | 10 | From causes unrelated to Knee surgery |
| Aged > 85 years at time of study | 13 | Outside the limits of the ethical agreement |
| Invited but declined | 4 | Invitation via telephone and/or email |
| Unable to contact | 6 | No response from telephone / email invitations (four patients live outside of the UK) |
